# Invited discussant comments during the UCL–Penn Global Covid Study webinar ‘Family Life: Stress, Relationship Conflict and Child Adjustment’

**DOI:** 10.14324/111.444/ucloe.100001

**Published:** 2022-09-16

**Authors:** Yahayra Michel

**Affiliations:** 1 School of Criminology and Justice Studies, University of Massachusetts Lowell, Lowell, MA, USA

**Keywords:** family stress, pandemic, parent–child relations, child adjustment, child adverse behaviour, parental depression, parental stress, Covid-19

## Abstract

The main objective of this article is to comment on the findings presented during the UCL–Penn Global Covid Study webinar, ‘Family Life: Stress, Relationship Conflict and Child Adjustment’ by Portnoy and colleagues. The study examined the ways in which family stress conflict has been affected by the coronavirus (Covid-19) pandemic. Informed by the transactional models of parent–child behaviour, the authors are specifically interested in exploring the effect of child adjustment on parental outcomes. The study, currently under consideration for publication, found that child emotional and conduct problems predicted changes in parental depression and stress during the early stages of the Covid-19 pandemic. Child hyperactivity predicted parental stress, but not depression. None of the child behaviour problems (emotional problems, conduct problems and hyperactivity) predicted parental relational conflict. This article discusses reasons why the study under consideration did not find a significant effect on relational conflict and posts questions that can be addressed in future studies.

## About the study

The UCL–Penn Global Covid Study^1^ launched in April 2020 is a 12-month longitudinal study of the impact of Covid-19 on social trust, mental health and physical health. In collaboration with six institutions from Italy, Singapore, the United States, China and the United Kingdom,^2^ the study looks at the short- and longer-term effects of Covid-19 on an individuals’ mental health and social relationships with others. Survey data were collected at three time-points: 17 April–14 July 2020 (Wave 1), 17 October–31 January 2021 (Wave 2) and 17 April–31 July 2021 (Wave 3).

## About the webinar

Held online between 2 June and 28 July 2021, the study group presented research data at five online webinars, as part of the UCL Global Engagement Fund sponsorship, to discuss the lessons learned and invited policy makers and other subject experts to speak on the policy relevance and implications of the study findings. The recorded comments from these discussions focusing on the policy relevance and implications of each academic article were recorded as discussant articles and are published in this journal to be read alongside the research article being discussed.

These discussant articles are reviewed by members of the Editorial Board before being published. It is hoped that these discussant articles, read alongside the academic articles, will provide more holistic understanding of the issues at hand, how findings may inform policies in the coming months and/or assist in future crisis management strategies and aid decision-making, in an open and transparent manner.

The study was pre-registered https://osf.io/4nj3g/ on 17 May 2021) and ethical approval was obtained from the University College London Institute of Education Ethics and Review Committee on 8 April 2020 (REC 1331).^1^

## Linked research article

The linked research article to this discussion article cited here has been published in *UCL Open: Environment* following open peer review and made freely available to read as an open access article. Additionally, all previous versions and peer review reports are freely available to read as open access preprint articles from the journal’s preprint server by following the below DOI link and navigating to the version history of the published research article. Readers can find more information about how peer review works in the journal at ucl.scienceopen.com.

Portnoy J, Bedoya A, Wong K. Child externalising and internalising behaviour and parental wellbeing during the Covid-19 pandemic. *UCL Open: Environment*. 2022;(4):11. Available from: https://doi.org/10.14324/111.444/ucloe.000040

## Recorded webinar

This discussion article comments on the findings presented during the following webinar that has been recorded and made freely available to readers to watch on-demand.

Summer Webinar 3 – Family Life: Stress, relationship conflict and child adjustment. #GlobalCovidStudy. Available from: https://www.youtube.com/watch?v=l3C34EzyaBQ

## Introduction

The UCL–Penn Global Covid Study *Family Life: Stress, relationship conflict and child adjustment,* focuses on studying family relationships during the coronavirus (Covid-19) pandemic. It specifically seeks to contribute to our understanding of the bi-directional relationship dynamics that occur between parents and their children, by focusing on the effects of child behaviour on parental outcomes.

The research team collected data on relationships, trust, empathy, conflict, parenting and stress using parents’ reports on a 30-minute online survey. Data were collected in eight languages between April 2020 and January 2021, which reflect three UK lockdowns and easing (April–July 2021). The current study reported on two waves of data. Data collection efforts were ongoing for the third wave as the findings were presented.

Studies conducted early-on during the pandemic period reported that hardships attributed to Covid-19 were associated with worsened parental mood and increased uncooperativeness by children [1]. This study adds to the body of knowledge by measuring changes in parental outcome across two waves of data. More specifically, the study evaluated the extent to which parental depression and stress changed from Wave 1 to Wave 2 as a function of child emotional problems, conduct problems and hyperactivity at Wave 1. Results were presented as part of a series of online webinars between June and July 2021. The webinar for the study under consideration was presented on 30 June 2021.

Adverse child behaviours are linked to marital relationship conflicts, poor parental mental health and reduced quality of parenting practices. This study adds to the body of knowledge by exploring these complicated family dynamics under unique social, relational and emotional conditions: a global pandemic. These conditions are such that children’s behaviours are worsening just as parents’ mental and emotional capacities to deal with their disruptive behaviour are waning.

## Discussant comments

The discussion surrounding this paper aroused a great deal of questions and recommendations for future studies, particularly surrounding the finding that child emotional problems, conduct problems and hyperactivity were not associated with relational conflict. This article focuses on highlighting some of the questions and future research recommendations that came up during those conversations.

### Sustained effects

The findings presented were based on two waves of data collected between April 2020 and January 2021, allowing us to evaluate the change in parental outcomes during the initial stages of the pandemic and six months later. There is also a need to investigate and report on the sustained effects of child adjustment on parental outcomes as families and other social institutions learn to navigate the challenges associated with the Covid-19 pandemic. Governmental restrictions have varied over time and across nations, but overall, they have softened. Schools and businesses have re-opened. Parents have gone back to work. How have these shifts affected family dynamics?

### Parental relational conflict

The study reported no significant effect of child behaviour on martial conflict, which was unexpected. Several points related to this finding were discussed including a potential measurement issue and a limitation of the sample. As it pertains to the measurement issue, relationship conflict was measured using items that reflected general areas of relationship conflict, as opposed to conflicts directly related to parenting practices, which are more likely to be linked to child misbehaviour. The issue related to the sample revolved around the fact that it was relatively normative. Child level of misbehaviour may not have reached the threshold necessary to see significant results we expected to it.

Family systems theory [2], and in particular the concept of ‘scapegoating’, was brought up as a potential theoretical framework for understanding why child emotional problems, conduct problems and hyperactivity did not predict parental relationship conflict. The paradoxical idea that once a family member is ‘scapegoated’ difficult family dynamics become stabilised may be at play here. We are living in a time of global uncertainty and anxiety, which uniquely impacts families with children. These data were collected at the very early stages of the pandemic. It is possible that as these data were collected, the families represented in this study were operating under survival mode and would resort to any and all psychological processes in order to find stability in the most uncertain of times.

### Controls versus categories

A good portion of the ideas shared during the discussion involved looking at differences by sub-categories of the sample such as parents’ sex and age. These variables were introduced as controls in the regression models and therefore held constant. It is possible, however, that when looking at these findings overall and/or if we artificially hold something as constant that in fact varies, we are unintentionally concealing interesting and/or significant results by sub-categories of the sample. It may be worth trying to figure out if the effect of child behaviour on parental relationship conflict depends on the parents’ age, sex or profession.

### Self-report data versus official statistics

One of the strengths of this study is that it is based on self-report data. In the United States, reports of suspected child abuse and neglect decreased substantially during quarantine. At first glance, this may seem like a positive social change perhaps attributed to families spending more time together. On the other hand, it could also reflect time away from mandated reporters due to school closures. The importance of self-report data to complement the official statistics cannot be overstated.

### A call to unify

One of the most important take-away points from this study, and in particular the transactional model framework that it employs, is that family relationship dynamics are complex and multi-directional. Responses to family conflict and stress, particularly during times of heightened anxiety and uncertainty need to be multi-faceted to have a positive effect on our families and children. We cannot operate in silos and we cannot do it alone. Social service agencies often have more work to do than they have resources to do it with. Forming collaborative relationships across agencies is more important than ever.

## Notes

1 Wong KK, Raine A. Covid-19: Global study of social trust and mental health. Available from: https://globalcovidstudy.com [Accessed: 11 November 2021].

2 Wong KK, Raine A. Covid19: Global social trust and mental health study. 2020. https://doi.org/10.17605/OSF.IO/FE8Q7. [Accessed: 11 November 2021].
